# Cost-Effectiveness Analysis of Chatbot-Supported Remote Patient Monitoring for Anticoagulation Management: Health Economic Evaluation Within a Pilot Crossover Trial

**DOI:** 10.2196/85430

**Published:** 2026-06-17

**Authors:** Ana Rita Santos, Filipa Sampaio, Federico Guede-Fernández, Julian Perelman, Ana Londral

**Affiliations:** 1 Nova National School of Public Health, Universidade Nova de Lisboa, Lisbon, Portugal; 2 Comprehensive Health Research Centre, Lisbon, Portugal; 3 Value for Health CoLAB, Lisbon, Portugal; 4 Uppsala Health Economics, Department of Public Health and Caring Sciences, Uppsala University, BMC, Husargatan 3, Uppsala, 751 22, Sweden, 46 793253038; 5 Department of Learning, Informatics, Management and Ethics, Karolinska Institutet, Stockholm, Sweden; 6 LIBPhys (Laboratory for Instrumentation, Biomedical Engineering and Radiation Physics), Nova School of Science and Technology, Universidade Nova de Lisboa, Lisbon, Portugal; 7 Department of Physics, NOVA School of Science and Technology, Universidade NOVA de Lisboa, Lisbon, Portugal

**Keywords:** digital health, economic evaluation, cost-effectiveness, lifecycle, remote patient monitoring, chatbot, anticoagulants

## Abstract

**Background:**

Digital health technologies (DHTs) are increasingly integrated into clinical practice, yet economic evaluations remain scarce, particularly in the early development stages. Within the NICE (National Institute for Health and Care Excellence) Evidence Standards Framework, Tier C DHTs comprise technologies with direct clinical implications and measurable health outcomes, for which robust economic evidence is essential. Early-stage assessments are particularly important to inform subsequent development, refinement, and adoption decisions across the digital health lifecycle.

**Objective:**

This study aims to explore the feasibility of integrating a full trial-based economic evaluation within an early-stage pilot comparing a chatbot-supported remote patient monitoring (RPM) solution for anticoagulation management with the standard of care (SOC).

**Methods:**

A cost-effectiveness analysis was performed alongside a pilot crossover trial among adult cardiac surgery patients receiving vitamin K antagonists. Participants were allocated to two 6-month sequences (SOC→RPM or RPM→SOC). The intervention consisted of a rule-based chatbot integrated with home-based international normalized ratio self-testing using portable coagulometers to support communication and therapy management. Effectiveness was measured as time in therapeutic range (TTR), and costs were estimated from the Portuguese National Health Service and a limited societal perspective over a 1-year horizon. The analysis 1 applied a within-patient cost-effectiveness approach to estimate incremental costs, incremental TTR, and incremental cost-effectiveness ratios. Uncertainty was explored through nonparametric bootstrapping (5000 replications) and deterministic sensitivity analyses. Complementary comparisons examined differences between sequences (analysis 2), between periods (analysis 3), and within each sequence (analysis 4).

**Results:**

A total of 19 patients were included in the analyses. In analysis 1, RPM improved anticoagulation control, with a mean within-patient increase of 10.43 percentage points in time in TTR. The mean incremental costs were €198.61 (€1=US $1.08) from the Serviço Nacional de Saúde perspective and €270.05 from the limited societal perspective. The corresponding incremental cost-effectiveness ratios were €19.03 and €25.88 per additional percentage point of TTR gained. Sensitivity analyses produced consistent estimates across parameter variations. Complementary analyses (2-4) suggested that RPM tended to be more cost-effective when implemented after the initial 6-month postoperative period.

**Conclusions:**

This proof-of-concept study demonstrates that a full trial-based economic evaluation can feasibly be embedded within an early-stage Tier C DHT. The intervention showed improved anticoagulation control alongside higher costs, providing initial insights into its cost-effectiveness profile. Positioned within the digital health evidence continuum, such assessments can function as a learning stage within the lifecycle. To address the persistent adoption-evidence gap, tier- and stage-aligned frameworks are needed to guide the economic evaluation of DHTs. This study contributes to that goal by providing a set of recommendations specifically for Tier C DHTs.

## Introduction

Digital health technologies (DHTs) are increasingly used in health care, offering opportunities to improve outcomes and enhance system efficiency [1,2]. Yet, health technology assessment agencies and payer bodies continue to face challenges in adapting their methods to evaluate these technologies effectively [3]. The lack of robust evidence on their real benefits and cost-effectiveness hinders decision-making and risks fostering the diffusion of low-value, short-lived solutions [2]. Traditional approaches to economic evaluation, primarily designed and used for pharmaceuticals, are often misaligned with the fast-paced development of DHTs [4,5]. This creates a paradox: without evidence, adoption is unlikely; without adoption, generating evidence is difficult [6]. Some pioneering countries, such as Belgium and France, have begun to integrate economic evaluation into the assessment of DHTs [7]. However, others, such as Germany, through its Digitale Gesundheitsanwendungen fast-track scheme, do not require a formal economic evaluation for initial listing [3,7]. This uneven landscape underscores the absence of harmonized guidance on the generation and application of economic evidence for DHTs.

To bridge this gap, both regulatory initiatives and academic research have contributed to the development of dedicated frameworks for the economic evaluation of DHTs. Our recent scoping review identified 26 such frameworks and revealed substantial heterogeneity across key dimensions; however, 2 main recommendations emerged [8]. First, DHTs should be differentiated by purpose and risk, as reflected in the NICE (National Institute for Health and Care Excellence) Evidence Standards Framework [9] tiers: Tier A (low-risk, efficiency-focused solutions), Tier B (health and well-being support tools), and Tier C (high-risk clinical interventions with direct implications for diagnosis or treatment). Second, evaluation should follow stage-based approaches aligned with the DHT lifecycle, as emphasized in several frameworks [10-14]. For example, the World Health Organization maturity model [10] outlines a continuum of early (preprototype, prototype, pilot), mid (demonstration), and advanced (scale-up, integration) stages. This lifecycle perspective underscores the iterative nature of digital health evaluation, where evidence is built progressively through repeated assessments using different methodologies [11,12].

Despite this convergence, limited guidance exists on which economic evaluation methods are most appropriate at each maturity stage or tier. Model-based approaches are often recommended for early development [11,15], but they depend on the primary collection of reliable and context-specific data that can only be obtained through pilot studies [11]. The pilot stage is, therefore, critical for generating early evidence on feasibility, usability, and initial clinical and economic value. Pilot studies serve as structured learning opportunities to optimize technology use, identify barriers, and inform decisions for subsequent evaluations in later stages and for scale-up [10,16].

This study presents a pilot-stage use case of a Tier C DHT, integrating a full economic evaluation within a crossover trial of chatbot-supported remote patient monitoring (RPM) for postoperative anticoagulation therapy with vitamin K antagonists (VKAs) [15]. VKAs remain indispensable for patients with mechanical heart valves and other indications, but their use is clinically challenging due to a narrow therapeutic window: underdosing increases thromboembolic risk, while overdosing raises the risk of bleeding [17-22]. Anticoagulation control is best assessed by the time in therapeutic range (TTR). It is defined as the proportion of time a patient’s international normalized ratio (INR)—a standardized measure of blood clotting time that reflects anticoagulation control—remains within the target range, typically 2.0‐3.0 for most indications [20,23]. An inverse correlation between TTR and hemorrhagic and thromboembolic complications has been established [24-26]. Accordingly, current clinical guidelines recommend maintaining a TTR above 70% to minimize adverse events [17,22], which requires frequent INR monitoring and timely dose adjustments. Under the standard of care (SOC), INR is usually measured in health care facilities every 3 to 4 weeks [20], creating logistical barriers that may compromise anticoagulation control [27].

To address these challenges, the pilot crossover study evaluated a rule-based chatbot integrated with home-based INR self-testing using portable coagulometers to strengthen communication between patients and health care professionals and support timely therapy management [15]. The intervention improved anticoagulation control, particularly when introduced after the unstable postoperative period, resulting in gains in TTR [15]. Furthermore, a time-driven activity-based costing (TDABC) analysis of this intervention provided an initial understanding of its resource use and cost structure [15]. Building on these clinical and economic findings, this study extends this work by conducting a within-trial cost-effectiveness analysis (CEA) of chatbot-supported RPM compared with SOC over a 1-year time horizon from the perspective of the Portuguese National Health Service (Serviço Nacional de Saúde, SNS) and a broader, albeit limited, societal perspective. By embedding a full CEA into a pilot stage, this study aims to explore the feasibility of integrating economic assessment into early-stage Tier C DHTs. It also aims to illustrate how such evaluations can generate proof-of-concept evidence to inform subsequent model-based analyses and adoption decisions across the digital health lifecycle.

## Methods

### Study Design and Participants

The economic evaluation used data collected as part of an efficacy pilot crossover trial (ClinicalTrials.gov NCT06423521), whose primary clinical, implementation, and cost-related results have been published elsewhere [15], and it followed the CHEERS (Consolidated Health Economic Evaluation Reporting Standards) statement [28] (Checklist 1). In summary, the trial was conducted at the Department of Cardiothoracic Surgery, Hospital de Santa Marta, Lisbon. Eligible participants were adult patients (≥18 y) who had been discharged following cardiac surgery and prescribed long-term oral anticoagulation therapy with VKAs. Participants were assigned to 1 of 2 intervention sequences: SOC followed by RPM (group 1: SOC→RPM) or RPM followed by SOC (group 2: RPM→SOC), with each phase lasting 6 months, resulting in a total follow-up of 12 months per patient.

A total of 27 patients were enrolled between April 2021 and April 2023; 26 completed both phases and were included in the pilot. The final sample comprised 13 women and 13 men, with a mean age of 55.7 (SD 13.3) years. For the present CEA, only patients with ≥2 INR measurements per phase were included to allow TTR calculation. The final analytic sample comprised 19 participants (n=9 group 1, n=10 group 2). A dropout analysis was conducted to compare baseline characteristics (age, sex, indication for anticoagulation therapy, and allocation group) between included and excluded participants.

### Interventions

During the SOC phase, participants received standard postoperative anticoagulation care, consisting of routine INR monitoring at health care facilities and in-person consultations for VKA dose adjustments.

In the RPM phase, participants self-monitored INR at home using a portable coagulometer (CoaguChek XS; Roche Diagnostics) and were supported by a digital platform with a rule-based chatbot enabling bidirectional communication with the clinical team. Patients received automated prompts via smartphone to report INR values and relevant symptoms, and any out-of-range results automatically triggered a clinical response, including dosage adjustments and rescheduling of measurements. The chatbot functioned as an intermediary, ensuring timely clinical review and continuity of VKA therapy management.

### Resource Use and Costs

Costs were estimated from 2 perspectives. The SNS perspective included intervention costs, health care resource use, and medication-related costs borne by the SNS. The SNS is a universal, predominantly tax-funded service that provides comprehensive care at minimal cost to patients, coexisting with a private sector that does not offer the same breadth of services.

We also considered the broader, albeit limited, societal perspective, encompassing, in addition, the health care and transportation costs borne by the patients. Productivity losses and other costs beyond the health sector were excluded, as these data were not collected during the trial. Value-added tax was applied following INFARMED’s (Autoridade Nacional do Medicamento e Produtos de Saúde, I.P. [National Authority of Medicines and Health Products]) guidelines [29] for the SNS perspective. For the limited societal perspective, value-added tax was also included based on existing literature [30,31].

Intervention-specific costs were attributed to the RPM phase and included the portable coagulometer (device), test strips, a one-time training session, and the provision of a chatbot platform. Costs for the coagulometer and test strips were obtained from the manufacturer (Roche). The device cost (€600, €1=US $1.08) was amortized over a 3-year lifetime [32] using the straight-line depreciation method, allocating one-sixth of the cost to each 6-month intervention, corresponding to €100 per patient. INR test costs were estimated by multiplying the number of patient tests by the unit price of strips. A one-time training session was assumed to be the unit price for a home visit according to the 2023 hospital financing contract [33], under the assumption that training involved comparable professional time [34]. The chatbot platform, developed and maintained internally by Value for Health CoLAB, was costed at €50 per patient for the 6 months, reflecting market prices of comparable low-code solutions. This approach was used as a proxy in the absence of detailed internal cost data and captures development, infrastructure, basic maintenance, and text messages.

Health care resource use included medical and nursing consultations, hospitalizations, emergency visits, INR testing (SOC phase), and anticoagulant medication. Resource quantities were obtained from patient records, with hospitalizations coded by Diagnosis-Related Groups (Grupos de Diagnóstico Homogéneo), which also provided unit costs by severity. Total health care costs per patient were then calculated by multiplying unit costs by the corresponding quantities of resources used. Unit costs for medical consultations and emergency visits were based on the 2023 hospital financing contract, which sets the prices to be paid to SNS hospitals for several services [33]. Nursing consultations, not included in the contract, were estimated at half the cost of a medical consultation, consistent with the ratio between medical and nursing consultation costs reported in Ordinance 207/2017 [35]. Hospitalizations and INR tests were costed using the same ordinance [35]. In line with national regulations [35], only hospitalization costs were considered when an emergency visit led to admission; otherwise, emergency visits were valued at the standard daily rate for medical-surgical emergency care. The cost of INR testing during the SOC phase was assumed to be identical across settings (primary care, hospital, or private laboratory). Medication costs were taken from the INFARMED database [36]. Given that dosage data were incomplete and prescribed doses of VKAs vary substantially according to INR values (typically ranging from 2.5 to 10 mg for warfarin [37] and 1 to 8 mg for acenocoumarol [38]), fixed daily doses of 5 mg warfarin and 4 mg acenocoumarol were applied as a simplifying and conservative assumption, reflecting typical midrange dosing in clinical practice. Medication costs were valued using the lowest available market price, and for the SNS perspective, patient co-payments corresponding to 31% of the retail price were excluded [26].

Travel costs were considered for in-person medical and nursing consultations and for INR testing during the SOC phase. Distances were calculated as round trips between patients’ residences and health care facilities. For mainland Portugal, personal car travel was assumed. For patients from Madeira and the Azores islands, local INR testing used car travel, while consultations held on the mainland assumed public transport. This distinction reflects the context of the islands, where local INR testing is done nearby, while travel to mainland hospitals requires air travel. Costs followed official reimbursement rates [39,40].

All costs were expressed in 2024 euros (€), adjusted using the Consumer Price Index [41], except for medication, which used 2025 INFARMED prices. As the time horizon was 1 year, no discounting was applied [29]. Unit costs and resources are summarized in Table 1.

**Table 1.** Unit costs, including value-added tax (2024 €, except for medication costs in 2025 €).

Cost item	Unit cost (€)	Source
Intervention costs (RPM^a^ only)		
Machine	600	Roche
Test strip	3.88	Roche
Training session	42.94	Hospital financing contract 2023
Chatbot platform	50	Own estimate
Health and health care use		
Medical consultations	80.76	Hospital financing contract 2023
Nurse consultations	40.38	Own estimate based on Hospital financing contract 2023 and Portuguese Ordinance 207/2017 of 11th July
Medical consultations (telemedicine)	88.94	Hospital financing contract 2023
Hospitalizations	Varies by GDH^b^	Portuguese Ordinance 207/2017 of 11th July
Emergency	111.43	Hospital financing contract 2023
INR^c^ test (SOC^d^ only)	3.42	Portuguese Ordinance 207/2017 of 11th July
Medication costs		
Warfarin	0.12/day (5 mg)	INFARMED
Acenocoumarol	0.17/day (4 mg)	INFARMED
Out-of-pocket costs		
Travel costs	0.46/km (personal car) 0.14/km (public transportation)	Portuguese Ordinance 1553-D/2008 of 31st December revised by Decree Law 137/2010 of 28th December

a RPM: remote patient monitoring.

b GDH: Grupos de Diagnóstico Homogéneo.

c INR: international normalized ratio.

d SOC: standard of care.

### Health Outcomes

The primary outcome was the difference in TTR between the SOC and RPM phases. TTR was defined as the percentage of time, measured in days, that a patient’s INR values remained within the target therapeutic range, typically between 2.0 and 3.0, depending on individual clinical indications. TTR was calculated for each intervention phase using the Rosendaal method of linear interpolation, which estimates the proportion of time spent within the therapeutic range based on successive INR values [42,43].

During the RPM phase, the expected frequency of INR self-testing was approximately once every 15 days, as outlined in the study protocol. Nonetheless, the clinical team could modify the testing schedule based on individual results. If a patient’s INR values were outside the therapeutic range, clinicians were permitted to increase the testing frequency to optimize therapeutic adjustment and reduce the time required to restore INR control.

### Statistical Analysis

Data management and costing were performed in Microsoft Excel (version 16.98), and statistical analyses were performed in RStudio (version 2025.05.1+513; Posit Software, PBC).

Descriptive statistics were used to summarize the data. Categorical variables were reported as counts and percentages, and continuous variables as mean (SD) or median (IQR), depending on the data distribution assessed by the Shapiro-Wilk test. For the analysis of patients’ characteristics and dropout comparison, Chi-square tests or Fisher exact tests were used for categorical variables, and independent *t* tests or Wilcoxon rank-sum tests for continuous variables, depending on the distribution of the data.

Mean differences in costs and TTR between intervention phases were analyzed using linear regression models, where the dependent variables were costs and TTR, and the main explanatory variable was the intervention phase (RPM vs SOC). No additional covariates were included. Statistical significance was defined as *P*<.05.

The analytical strategy comprised 4 components. Analysis 1 consisted of the base-case within-patient CEA, comparing RPM with SOC over a 1-year time horizon from both the SNS and limited societal perspectives. Analysis 2 involved a between-sequence comparison, contrasting the 2 crossover sequences (SOC→RPM vs RPM→SOC). Analysis 3 included period-specific comparisons, contrasting SOC in Sequence 1 with RPM in Sequence 2 during Period 1, and RPM in Sequence 1 with SOC in Sequence 2 during Period 2. Analysis 4 comprised within-sequence comparisons, assessing RPM vs SOC separately within each sequence according to the order in which participants received the interventions.

For the base-case analysis 1, the incremental cost-effectiveness ratio (ICER) was calculated as the mean difference in costs divided by the mean difference in TTR (percentage points), representing the additional cost per unit improvement in anticoagulation control. Uncertainty around the ICER was assessed only for the base-case analysis using nonparametric bootstrapping (5000 iterations), which does not rely on distributional assumptions for costs or effects. This procedure provided 95% CIs for incremental costs, incremental TTR, and the ICER. The bootstrapped estimates were used to construct cost-effectiveness planes and cost-effectiveness acceptability curves (CEACs). The planes display the distribution of incremental cost-effect pairs across quadrants, where each quadrant has a decision-making implication, while CEACs illustrated the probability that RPM is cost-effective across a range of willingness-to-pay thresholds per additional percentage point of TTR.

### Sensitivity Analysis

One-way deterministic sensitivity analyses were conducted for the analysis 1 to assess the impact of key cost and effectiveness assumptions on the ICER. The parameters varied were: (1) chatbot cost (€25-€75/patient), reflecting system development, maintenance, and SMS text messaging usage; (2) coagulometer amortization, assuming lifespans of 5 and 10 years instead of the base-case 3 years; (3) travel distances (±20%) to account for geographic variability; and (4) TTR gains, using the lower and upper bounds of the 95% CI.

### Ethical Considerations

Ethical approval was obtained from the Ethics Committee of the ULS São José (REC number: 1057/2021) and was registered on ClinicalTrials.gov under the identifier NCT06423521.

## Results

### Participants’ Characteristics

**Figure 1.**
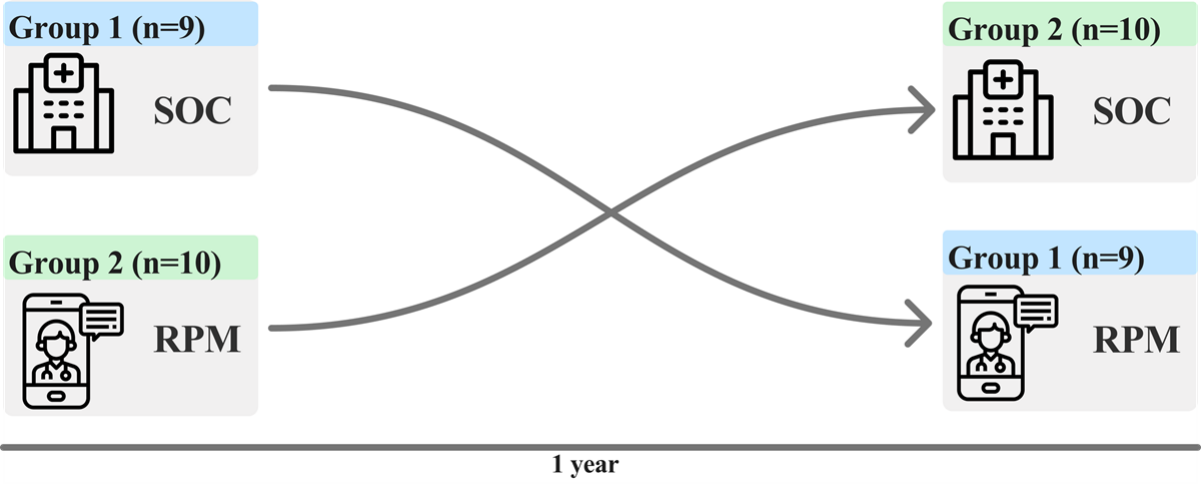
Flow diagram of the pilot crossover trial. SOC: standard of care; RPM: remote patient monitoring.

Demographic characteristics were well balanced between the 2 groups at baseline [15]. Table 2 summarizes the characteristics of the 19 participants included in the economic analysis (Figure 1). No statistically significant differences were observed between the groups in terms of age (*P=.*61) or sex (*P*=.31).

**Table 2.** Participants’ characteristics.

Variable	Overall (N=19)	Group 1 (n=9)	Group 2 (n=10)
Age (y), mean (SD)	55.37 (14.06)	53.56 (14.93)	57.00 (13.83)
Sex, female, n (%)	9 (47.37)	3 (33.33)	6 (60)
Indication for anticoagulation therapy, n (%)			
Mechanical aortic prosthesis	11 (57.89)	7 (77.78)	4 (40.00)
Pulmonary thromboembolism	2 (10.53)	1 (11.11)	1 (10.00)
Atrial fibrillation	1 (5.26)	1 (11.11)	0 (0.00)
Mitral valvuloplasty	1 (5.26)	0 (0.00)	1 (10.00)
Bentall procedure	1 (5.26)	0 (0.00)	1 (10.00)
Mechanical mitral prosthesis	2 (10.53)	0 (0.00)	2 (20.00)
Mechanical mitral prosthesis and atrial fibrillation	1 (5.26)	0 (0.00)	1 (10.00)
Number of INR^a^ measures, mean (SD)			
RPM^b^	21.21 (5.64)	18.44 (3.88)	23.7 (5.98)
SOC^c^	11.32 (5.71)	15.33 (5.72)	7.7 (2.31)

a INR: international normalized ratio.

b RPM: remote patient monitoring.

c SOC: standard of care.

A dropout analysis comparing the 19 participants included in the TTR-based analysis with the 7 participants excluded due to insufficient INR data revealed no statistically significant differences in baseline characteristics. Specifically, there were no significant differences in sex distribution (Fisher exact test, *P*>.99), age (Welch *t* test, *P*=.89), indication for anticoagulation (Fisher exact test, *P*=.38), or intervention sequence group (Fisher exact test, *P*=.66). These findings suggest that dropout is unlikely to have introduced bias into the analysis.

### Resource Use and Costs

Detailed mean costs per participant by category and perspective are presented in Table 3. Intervention-specific items (coagulometer, test strips, chatbot platform, and training) contributed exclusively to the RPM phase, accounting for €275 per patient on average. Among health care services, medical consultations were more frequent, with more associated costs during RPM (mean €191.27, SD €94.07) than SOC (mean €136.02, SD €137.40), while nurse consultations showed similar costs across phases (mean €146.64, SD €76.39 vs mean €148.77, SD €187.02). One patient required hospitalization during the SOC phase, contributing to an observed mean cost of €109.78 (SD €478.53). Medication costs were identical across phases and perspectives, and INR testing costs applied only to SOC (mean €38.70, SD €19.52). From the limited societal perspective, travel costs were higher during the RPM phase (mean €227.64, SD €295.75) than during SOC (mean €102.88, SD €129.88), likely reflecting the greater number of in-person medical consultations observed in the RPM phase.

**Table 3.** Costs (in €) per participant over the trial from the Serviço Nacional de Saúde and limited societal perspectives.^a^

Cost item	RPM^b^ phase, mean (SD)	SOC^c^ phase, mean (SD)	Mean difference (95% CI), *P* value
Intervention costs (RPM^b^ only) (both perspectives)			
Machine	100	—^d^	100
Test strip	82.27 (21.89)	—^d^	82.27
Training session	42.94	—^d^	42.94
Chatbot platform	50	—^d^	50
Health and health care use (both perspectives)			
Medical consultations	191.27 (94.07)	136.02 (137.40)	55.26 (−19.62 to 130.13), *P*=.16
Nurse consultations	146.64 (76.39)	148.77 (187.02)	−2.13 (−92.96 to 88.71), *P*=.96
Medical consultations (telemedicine)	18.72 (63.44)	0	18.72
Hospitalizations	0	109.78 (478.53)	−109.78
Emergency	0	0	0
INR^e^ costs (SOC^b^ only)	—^d^	38.70 (19.52)	−38.70
Medication costs (SNS^f^ perspective)			
Warfarin/Acenocoumarol	19.32 (3.44)	19.32 (3.44)	0
Medication costs (limited societal perspective)			
Warfarin/Acenocoumarol	27.76 (4.30)	27.76 (4.30)	0
Out-of-pocket costs (limited societal perspective)			
Travel costs INR^e^ (SOC^c^ only)	—^d^	53.28 (86.60)	−53.28
Other travel costs	227.64 (295.75)	102.88 (129.88)	124.71 (−20.53 to 269.96), *P*=.10

a Mean differences were calculated as RPM minus SOC; negative values indicate lower costs in the RPM phase.

b RPM: remote patient monitoring.

c SOC: standard of care.

d Not applicable.

e INR: international normalized ratio.

f SNS: Portuguese National Health Service (Serviço Nacional de Saúde).

Under the within-patient base-case comparison (analysis 1), mean costs were higher during RPM than during SOC. From the SNS perspective, the mean difference was €198.61 (95% CI –€90.19 to €487.41; *P*=.19), while from the limited societal perspective, the difference was €270.05 (95% CI –€75.29 to €615.39; *P*=.13).

When the 2 crossover sequences were contrasted (analysis 2), costs were lower in the RPM→SOC sequence. The estimated differences were –€108.30 from the SNS perspective (95% CI –€402.55 to €186.04; *P*=.48) and –€191.00 from the limited societal perspective (95% CI –€542.43 to €160.43; *P*=.29).

Period-specific comparisons (analysis 3) showed that, in period 1, RPM was associated with incremental costs of €74.10 (95% CI –€443.29 to €591.50; *P*=.78) from the SNS perspective and €58.77 (95% CI –€524.15 to €641.69; *P*=.85) from the limited societal perspective. In Period 2, incremental costs increased substantially, reaching €290.61 (95% CI €95.32 to €485.90; *P*=.01) from the SNS perspective and €440.80 (95% CI €152.41 to €729.14; *P*=.01) from the limited societal perspective.

Finally, the within-sequence contrasts (analysis 4) indicated that, in the SOC→RPM sequence, RPM was associated with lower costs from the SNS perspective (–€126.47, 95% CI –€672.97 to €420.04; *P*=.63) but considerably higher costs from the limited societal perspective (€491.18, 95% CI €304.87 to €677.49; *P*<.001). In the opposite sequence (RPM→SOC), RPM again had lower costs from the SNS perspective (–€135.47, 95% CI –€726.57 to €455.62; *P*=.66) and higher costs from the limited societal perspective (€635.02, 95% CI €320.51 to €949.52; *P*<.001). All incremental cost estimates for analyses 1-4 are detailed in Table 4.

**Table 4.** Incremental costs and incremental effects across analyses 1-4 from the Serviço Nacional de Saúde and limited societal perspectives.^a^

Analysis and outcome	SNS^b^ perspective: incremental costs, € (95% CI), *P* value	Limited societal perspective: incremental costs, € (95% CI), *P* value	Incremental effects (TTR^c^), percentage points, (95% CI), *P* value
Analysis 1: within patient comparison			
Base-case analysis	198.61 (−90.19 to 487.41), .19	270.05 (−75.29 to 615.39), .13	10.43 (−3.74 to 24.61), .16
Analysis 2: between sequence comparison			
SOC^d^→RPM^e^ vs RPM^e^→SOC^d^	−108.30 (−402.55 to 186.04), .48	−191.00 (−542.43 to 160.43), .29	−8.98 (−23.28 to 5.32), .23
Analysis 3: period specific comparisons			
Period 1: RPM (RPM^e^→SOC^d^) vs SOC (SOC^d^→RPM^e^)	74.10 (−443.29 to 591.50), .78	58.77 (−524.15 to 641.69), .85	2.06 (−12.94 to 17.05), .79
Period 2: RPM (SOC^d^→RPM^e^) vs SOC^d^ (RPM^e^→SOC^d^)	290.61 (95.32 to 485.90), .01	440.80 (152.41 to 729.14), .01	20.02 (−3.05 to 43.09), .11
Analysis 4: within sequence comparisons			
RPM^e^ vs SOC^d^ (SOC^d^→RPM^e^)	−126.47 (−672.97 to 420.04), .63	491.18 (304.87 to 677.49), <.001	22.53 (7.34 to 37.71), .01
RPM^e^ vs SOC^d^ (RPM^e^→SOC^d^)	−135.47 (−726.57 to 455.62), .66	635.02 (320.51 to 949.52), <.001	−0.45 (−22.65 to 21.75), .97

a Incremental values are reported as RPM compared with SOC. Negative cost differences indicate lower costs with RPM, and positive values for incremental effects (TTR, percentage points) indicate greater improvement in anticoagulation control.

b SNS: Portuguese National Health Service (Serviço Nacional de Saúde).

c TTR: time in therapeutic range.

d SOC: standard of care.

e RPM: remote patient monitoring.

### Health Outcomes

In the base-case within-patient comparison (analysis 1), mean TTR was higher during RPM (60.70%, SD 17.50%) than during SOC (50.26%, SD 26.21%), yielding an incremental difference of 10.43 percentage points (95% CI –3.74 to 24.61; *P*=.16).

When the 2 crossover sequences were compared (analysis 2), TTR was, on average, 8.98 percentage points higher in the SOC→RPM sequence than in the RPM→SOC sequence, although this difference did not reach statistical significance (95% CI –5.32 to 23.28; *P*=.23).

Period-specific contrasts (analysis 3) showed a small and nonsignificant incremental effect in period 1 (2.06 percentage points, 95% CI –12.94 to 17.05; *P*=.79), and a larger but still nonsignificant difference in period 2 (20.02 percentage points, 95% CI –3.05 to 43.09; *P*=.11).

Finally, the within-sequence comparisons (analysis 4) indicated that participants in the SOC→RPM sequence experienced a statistically significant improvement in TTR during the RPM phase relative to SOC (22.53 percentage points, 95% CI 7.34 to 37.71; *P*=.01). No significant change was observed in the RPM→SOC sequence, where the difference was –0.45 percentage points (95% CI –22.65 to 21.75; *P*=.97). All incremental effectiveness estimates for analyses 1-4 are detailed in Table 4.

### Cost-Effectiveness Analysis

**Figure 2.**
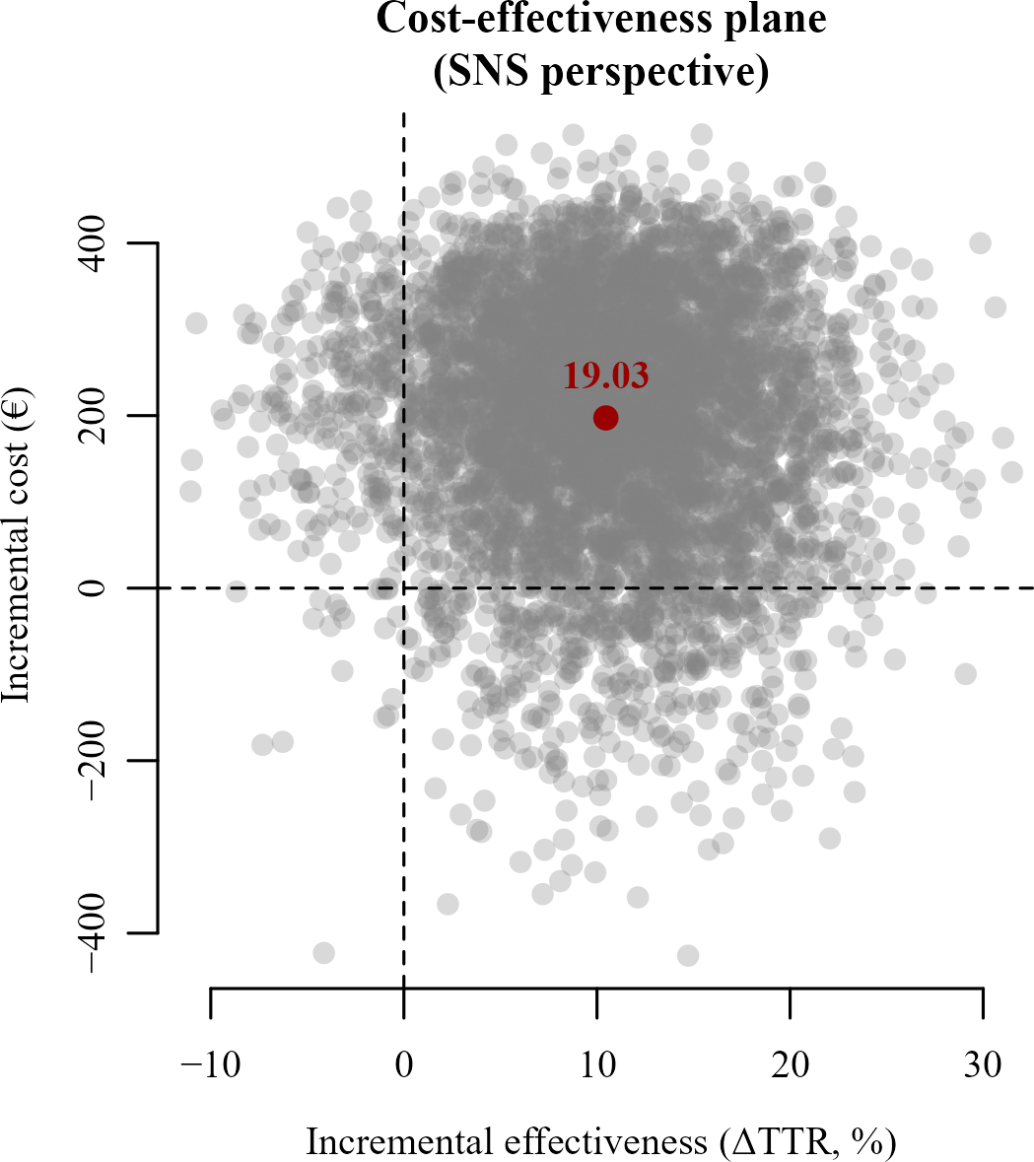
Cost-effectiveness plane (Serviço Nacional de Saúde [SNS] perspective). TTR: time in therapeutic range.

**Figure 3.**
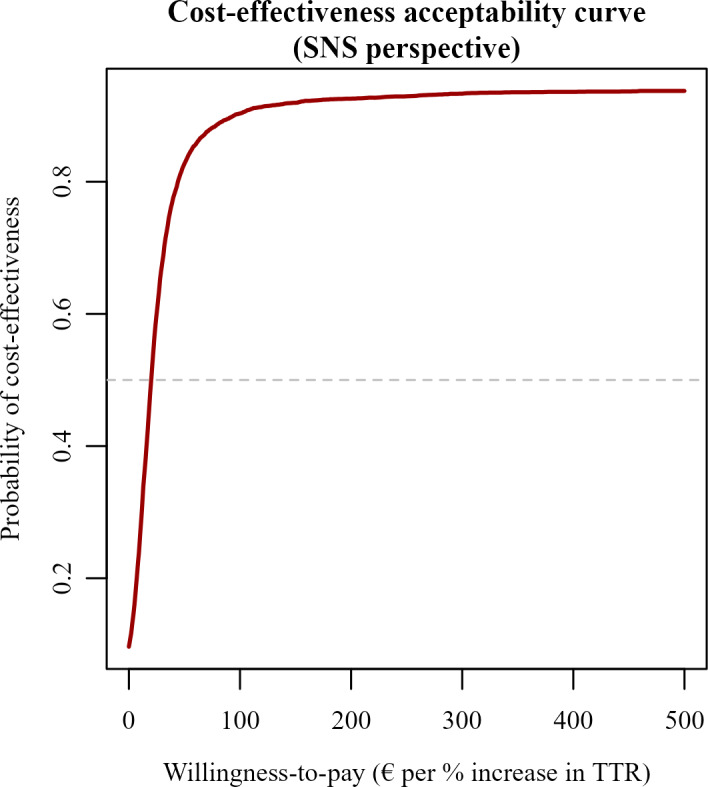
Cost-effectiveness acceptability curve for the Serviço Nacional de Saúde (SNS) perspective. TTR: time in therapeutic range.

From the SNS perspective, the base-case ICER was €19.03 per additional percentage point of TTR gained with RPM compared with SOC. Uncertainty was further explored through nonparametric bootstrapping (5000 replications). The cost-effectiveness plane (Figure 2) illustrates the distribution of incremental cost-effectiveness pairs. Most estimates (4275/5000, 85.5%) fell within the northeast quadrant, indicating that RPM was more effective and more costly than SOC; 9.1% (455/5000) were in the southeast quadrant, suggesting improved effectiveness at lower costs; 5% (250/5000) were in the northwest (higher cost, lower effectiveness); and 0.4% (20/5000) were in the southwest (less effective and less costly). The CEAC (Figure 3) shows that, at a willingness-to-pay (WTP) of €95 per percentage TTR gained, the probability of cost-effectiveness reached 90%.

**Figure 4.**
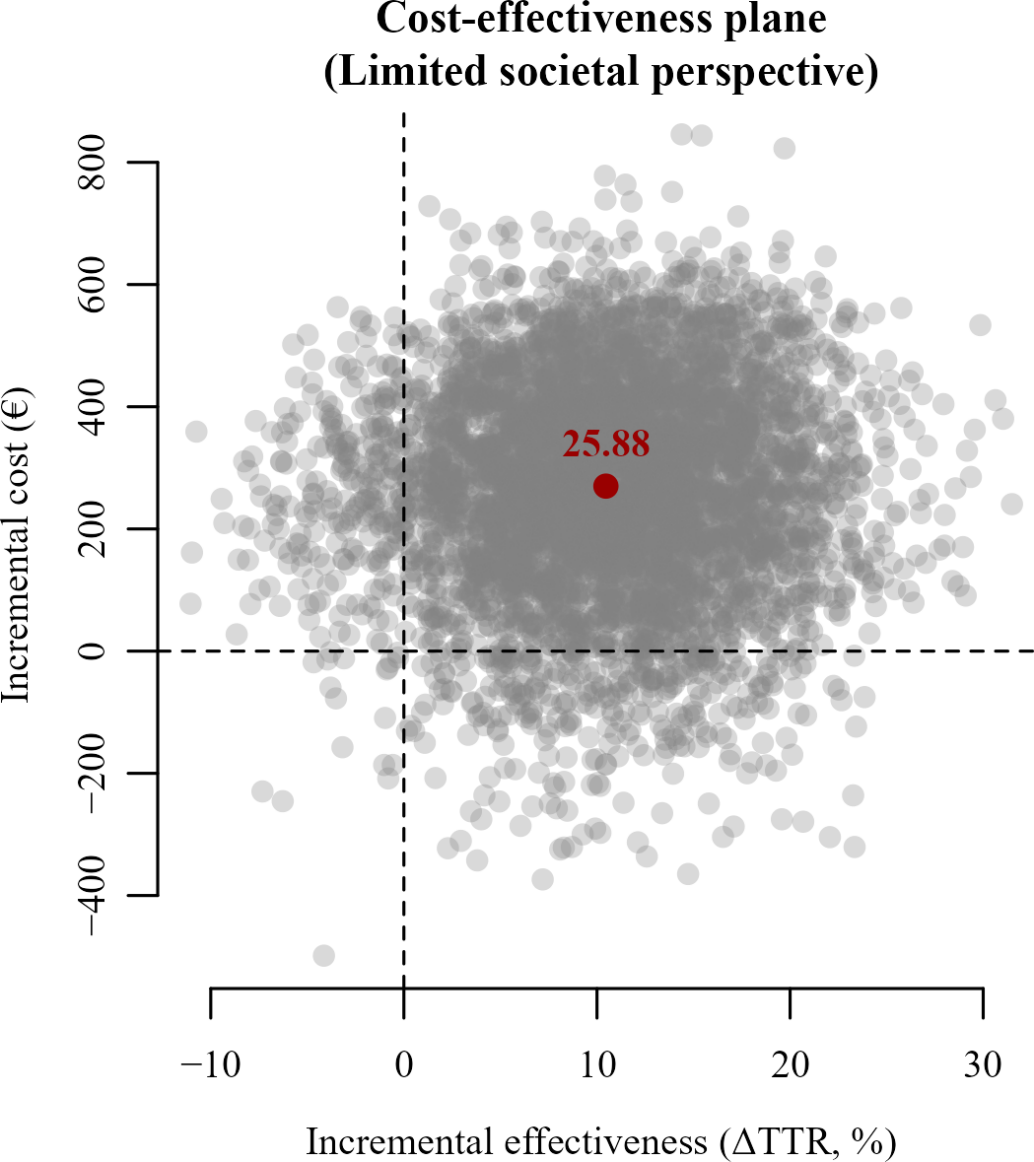
Cost-effectiveness plane (limited societal perspective). TTR: time in therapeutic range.

**Figure 5.**
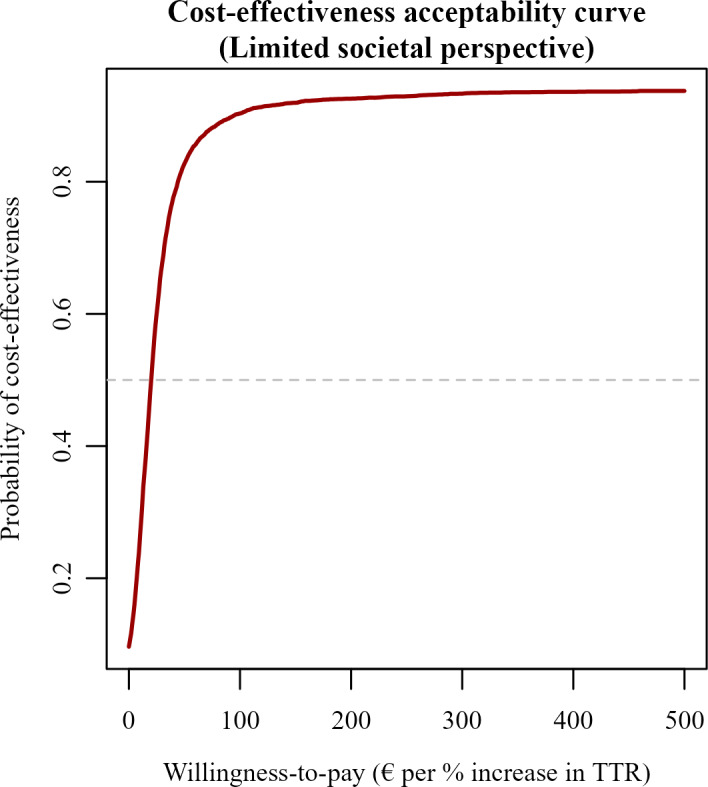
Cost-effectiveness acceptability curve for the limited societal perspective. TTR: time in therapeutic range

From the limited societal perspective, the base-case ICER was €25.88 per percentage point of TTR gained. The corresponding cost-effectiveness plane (Figure 4) showed a similar pattern, with 88% (4400/5000) of bootstrapped replications in the northeast quadrant, 6.5% (325/5000) in the southeast, 5% (250/5000) in the northwest, and 0.4% (20/5000) in the southwest. The CEAC (Figure 5) likewise indicated a 90% probability of cost-effectiveness at a WTP threshold of about €95 per percentage TTR gained.

### Sensitivity Analysis

One-way deterministic sensitivity analyses are summarized in Table 5. Varying the cost of the chatbot between €25 and €75 per patient shifted the ICERs between €16.65 and €21.44 per percentage point of TTR gained from the SNS perspective, and between €23.49 and €28.29 from the limited societal perspective. Extending the useful lifetime of the coagulometer from 3 to 5 or 10 years reduced the ICERs to €15.21 and €12.33 (SNS) and €22.06 and €19.18 (societal), respectively. Altering travel costs by ±20% had only a modest impact on the societal ICERs (ranging from €24.52 to €27.26). When the lower bound of the 95% CI for ΔTTR (–3.74) was applied, RPM was dominated (more costly and less effective). Conversely, using the upper bound (24.61 percentage points) substantially improved cost-effectiveness, lowering ICERs to €8.07 (SNS) and €10.97 (societal).

**Table 5.** Results of the sensitivity analysis from the Serviço Nacional de Saúde and limited societal perspectives.^a^

Scenario	ICER^b^ SNS^c^ perspective	ICER^b^ limited societal perspective
Analysis 1: base-case	19.03	25.88
Chatbot costs €25	16.65	23.49
Chatbot costs €75	21.44	28.29
Machine lifetime 5 y	15.21	22.06
Machine lifetime 10 y	12.33	19.18
Travel costs −20%	—^d^	24.52
Travel costs +20%	—^d^	27.26
ΔTTR^e^ low (95% CI)	Dominated	Dominated
ΔTTR[Table-fn T5_FN5] higher (95% CI)	8.07	10.97

a ICERs are reported as the incremental cost per percentage point increase in TTR, comparing RPM with SOC. “Dominated” indicates that RPM is more costly and less effective than SOC.

b ICER: incremental cost-effectiveness ratio.

c SNS: Portuguese National Health Service (Serviço Nacional de Saúde).

d Not applicable.

e TTR: time in therapeutic range.

## Discussion

### Principal Findings

This proof-of-concept study demonstrates the feasibility and methodological value of embedding a full trial-based economic evaluation within an early-stage Tier C DHT. Rather than seeking to establish definitive cost-effectiveness, it examined whether a full economic evaluation could be integrated into a small-scale clinical study to generate actionable evidence under real-world conditions. The resulting data provide an empirical foundation for subsequent model-based analyses and support evidence-informed adoption decisions along the digital health lifecycle.

From both the SNS and limited societal perspectives, the chatbot-supported RPM solution incurred higher costs (€198.61 and €270.05, respectively) while improving anticoagulation control, with a mean within-patient gain of 10.43 percentage points in TTR. The corresponding ICERs were €19.03 and €25.88 per additional percentage point of TTR gained, respectively. As no established WTP thresholds exist for percentage-point improvements in TTR, interpretation of cost-effectiveness remains challenging. Nevertheless, the relatively low ICERs observed suggest that clinically meaningful improvements in anticoagulation control may be achieved at a modest additional cost. This is particularly relevant given previous evidence from Wan et al [26], showing that increases of 8.3% and 10.2% in TTR were associated with approximately 1 fewer major hemorrhage and thromboembolic event per 100 patient-years, respectively. In this context, the CEACs indicate that at approximately €95 per percentage TTR gained, the probability of cost-effectiveness reaches 90%, further supporting the potential economic value of the intervention.

These findings indicate where early value begins to emerge and what drives it: intervention-specific components (coagulometer, test strips, and chatbot platform) and higher follow-up intensity contributed most to incremental costs, whereas greater therapeutic stability after the 6-month postoperative period was associated with improved control. Sensitivity analyses indicated that these results were generally robust across variations in key parameters.

Analyses 2-4 helped contextualize these findings by showing that implementation timing played an important role. Differences between sequences and periods indicated that RPM tended to be more effective and potentially more cost-effective once patients had progressed beyond the immediate postoperative phase. These patterns suggest that clinical stability may be a key condition under which the intervention generates greater value.

Taken together, the base-case cost-effectiveness results, supported by sensitivity analyses and analyses (2-4), illustrate how early-stage full economic evaluations can move beyond partial assessments to provide structured learning. Such insights demonstrate not only how much an intervention costs but also for whom and under which conditions it begins to generate value, thereby informing both future economic evaluations and decisions regarding the development, refinement, and adoption of these technologies.

### Comparison With Prior Work

Previous trial-based economic evaluations of self-monitoring interventions in anticoagulation management have also reported increased costs [27,32,44]. However, only 1 used TTR as the primary outcome, reporting significantly higher TTR values compared with routine care (72%, SD 19.7% vs 59%, SD 13.5%), alongside an incremental cost of €59.08 per patient over 6 months, although no ICER was reported [44]. To our knowledge, this is the first trial-based economic evaluation of a chatbot integrated into RPM for anticoagulation therapy. The cost-effectiveness of such digital health tools remains largely unexplored. A 2022 systematic review identified only 2 economic evaluations of chatbot-based interventions and emphasized the urgent need for robust, well-reported analyses in this rapidly evolving field [45]. This is particularly relevant, as the use of chatbots within RPM offers new opportunities to enhance health care delivery, strengthen patient-provider communication, and advance patient-centered care [46].

From a lifecycle perspective, this study represents the next analytical step for this specific RPM solution and, more generally, for Tier C DHTs. The earlier TDABC analysis served as a partial economic evaluation**,** offering early feasibility insights through the mapping of patient pathways, quantification of resource use, and identification of key cost drivers related to equipment, staff, and workflow requirements [15]. Such analyses are valuable for understanding cost structures, anticipating resource needs, and laying the groundwork for more comprehensive evaluations [11]. The current trial-based full economic evaluation, based on primary data, extends that groundwork by adding comparative value evidence, informing refinement of the RPM solution, supporting the design of the demonstration-phase study, and providing the necessary parameter estimates for subsequent model-based economic evaluations. Positioned within this continuum, the study supports the methodological argument that trial-based full economic evaluations can function as a learning stage in the digital health lifecycle.

### Limitations

The findings of this study should be interpreted with caution due to several limitations. The small sample size, associated nonsignificant results, single-center setting, and short follow-up period were expected features of a pilot stage but limit the interpretability and generalizability. Accordingly, the evidence should be considered hypothesis-generating rather than confirmatory.

From a design perspective, the crossover format may have introduced residual carry-over effects. Although analyses 2-4 were conducted to explore differences related to treatment order and timing, results were not fully consistent, and some variation between periods and sequences was observed. Therefore, carry-over effects cannot be excluded and may have influenced the estimation and interpretation of both outcomes and costs.

From an evaluation perspective, resource-use data were obtained exclusively from electronic health records at ULS São José, which may not capture health care use outside this institution. Moreover, because the economic evaluation was not prospectively planned, important data were not collected. Productivity costs were absent, precluding the adoption of a broader societal perspective. In addition, patient-reported outcomes, such as EQ-5D, were not collected, which prevented the estimation of quality-adjusted life years (QALYs). This limitation is particularly relevant for Tier C DHTs, which directly affect health outcomes, and evaluations should include QALY measurement to enable comparability across interventions for resource allocation decisions. However, TTR, as a clinically relevant measure of anticoagulation control, reflects the quality and effectiveness of anticoagulation management and allows comparability across similar interventions within this clinical area.

Furthermore, several aspects of the findings are context-specific, including unit costs, resource use patterns, and care pathways within the Portuguese health care setting. However, insights related to implementation timing, cost drivers, and the feasibility of embedding economic evaluation within early-stage studies may be transferable to similar contexts.

Taken together, these limitations provide valuable methodological lessons for Tier C DHTs. They highlight the need for prospective planning of economic end points, integration of QALYs, and alignment between clinical and economic data collection. In this sense, the limitations of the study are themselves instructive, illustrating the iterative learning inherent to early lifecycle economic evaluations.

### Recommendations for a Lifecycle Economic Evaluation Framework for Tier C DHTs

In response to the identified gap in applying suitable economic evaluation methods across maturity stages, and drawing on the evidence generated in this pilot stage, together with its main limitations (the absence of a prospectively planned economic evaluation and the lack of EQ-5D and productivity-loss data) and further complemented by prior literature [8,11,16,47,48], several recommendations are proposed to guide the development of a lifecycle-aligned framework for the economic evaluation of Tier C DHTs:

1. Embed economic evaluation throughout the lifecycle. Economic evaluation should be viewed as an iterative process that evolves in parallel with the technology’s maturity. For Tier C interventions, early stages (preprototype, prototype, pilot) should focus on feasibility and process costs through partial economic evaluations, such as TDABC, while also beginning to assess comparative effectiveness and uncertainty to inform subsequent stages. Mid stages (demonstration) should consolidate evidence on effectiveness and cost drivers, and advanced stages (scale-up and integration) should address long-term value and population impact through model-based approaches.
2. Plan the economic evaluation prospectively. Economic end points should be defined early and integrated from the outset. Prospective planning enables systematic tracking of costs and outcomes, ensures methodological alignment between clinical and economic components, and minimizes the risk of missing data that could compromise subsequent analyses.
3. Align clinical and economic outcomes. For Tier C DHTs, outcome measurement must capture both clinical and patient-reported outcomes. Instruments such as the EQ-5D should be collected alongside condition-specific indicators to enable QALY estimation, facilitate cost-utility analysis, and improve comparability across interventions and disease contexts.
4. All relevant cost categories (intervention-specific, health care payer, patient/caregiver, and productivity costs) should be defined and measured from the outset to enable a broader societal perspective and to capture the broader economic impact of Tier C DHTs.
5. Promote multidisciplinary and stakeholder collaboration. Robust economic evaluation of Tier C DHTs requires close collaboration among health economists, clinicians, digital-health developers, data scientists, and policymakers. Early and sustained engagement of these groups ensures methodological consistency, contextual relevance, and meaningful translation of findings into the development, pricing, and decisions regarding the adoption of these technologies.

Together, these recommendations outline the core principles for structuring a lifecycle economic evaluation framework for Tier C DHTs. Embedding economic evaluation across all maturity stages enables more coherent, comparable, and value-based evidence generation.

### Conclusion

This Tier C proof-of-concept study demonstrates that a full trial-based economic evaluation can feasibly be embedded within an early-stage DHT, producing actionable comparative evidence under real-world conditions. The intervention resulted in improved anticoagulation control alongside higher costs, providing early insights into its cost-effectiveness profile within a pilot setting. Beyond these numerical results, the study illustrates how such evaluations operate as structured learning experiments, identifying cost and effect drivers, testing methodological feasibility, and informing model-based projections and implementation readiness.

Building on the preceding TDABC analysis, this study exemplifies the role of pilot-stage evaluations within the digital health evidence continuum. Looking forward, tier- and stage-aligned frameworks are needed to strengthen this continuum and address the persistent adoption-evidence gap. This study contributes to that goal by providing a set of recommendations specifically for Tier C DHTs, supporting more consistent and value-based economic evaluations across the digital health lifecycle.

## Supplementary material

10.2196/85430 Checklist 1 CHEERS 2022 checklist.

## Abbreviations

CEA: cost-effectiveness analysis
CEAC: cost-effectiveness acceptability curve
CHEERS: Consolidated Health Economic Evaluation Reporting Standards
DHTs: digital health technologies
ICER: incremental cost-effectiveness ratio
INFARMED: Autoridade Nacional do Medicamento e Produtos de Saúde, I.P. [National Authority of Medicines and Health Products]
INR: international normalized ratio
NICE: National Institute for Health and Care Excellence
QALYs: quality-adjusted life years
RPM: remote patient monitoring
SNS: Serviço Nacional de Saúde [Portuguese National Health Service]
SOC: standard of care
TDABC: time-driven activity-based costing
TTR: time in therapeutic range
VKAs: vitamin K antagonists
WTP: willingness-to-pay
